# The Research Hospital at Cambridge

**Published:** 1908-12-19

**Authors:** Clifford Allbutt

**Affiliations:** St. Radegvnd's, Cambridge


### "THE RESEARCH HOSPITAL AT CAMBRIDGE."

*To the Editor of* The Hospital.

Sir,—Permit me to thank you for the generous words of encouragement in which you spoke last week of our venture in clinical research; in our appeal for funds your countenance is much valued by us. But you ask why we do not make these researches in Addenbrooke's Hospital and, as you go on to say, in my ward? To answer this question will, with your permission, make our purpose clearer, a purpose apart from ordinary hospital work. Perhaps we might have used some other name for our small institution, but one equally significant would not be easy to find. An ordinary hospital, such as Addenbrooke's, is for the treatment of patients; in our research hospital treatment is not the main purpose, as the patients themselves are well aware when they come in. They come in to be investigated. It is turning out that by research remedial lines of work are being discovered which are bringing welcome aid to many of the sufferers; but this is a recent and incidental result, for the most part such methods would be carried out at home, by their own physicians. Again, a general hospital is a charity; our research is no charity. No questions are asked as to the means of patients. The only inquiry is as to the nature of the malady—whether it is a good example of that which we are studying. Many persons in sufficient or even easy circumstances offer themselves for research, in the noble desire to render an indirect service to future sufferers. It is a matter of satisfaction to us that in this appeal considerable sums have been sent to us by patients who had previously shown their generosity in submitting to our investigation. One patient, whose case had been closely studied here, has sent us a hundred pounds, and other very generous donations from previous inmates have come in. Furthermore, many of the patients come from long distances, from physicians who send them to us for investigation. We cannot ask the Addenbrooke's subscribers to pay for these patients, nor to alienate beds for them. Moreover, these are chronic cases which no general hospital receives except at some crisis of their malady, whereas we want them at times of relative quiescence when they can best tolerate the attentions of our scientists. Then in what general hospital would it be practicable to allow bacteriologists, chemists, $x$-ray operators, and other investigators and annotators, most of whom have no connection with the hospital, to have access to the wards at their convenience, for long inquiries, and with cumbrous apparatus, etc.? The ordinary hospital visits, teaching, nursing, cleaning, and other routine would be seriously interfered with. I may add that the governing bodies of general hospitals consist preponderantly and often exclusively of laymen, the evil consequences of which partiality I ventured to demonstrate in my recent address at Manchester. This ought not to be, but so it is; for instance, at Addenbrooke's the honorary staff is, as such, wholly unrepresented on the governing body. Now in the research hospital we have our own way, unembarrassed by imperfect sympathies.

In conclusion, sir, I thank you for your reference to myself, but I cannot let it be supposed that in this movement I have more than an official credit, and the credit of an ardent sympathy and belief in the methods now initiated and, I hope, to be put presently on a firm basis for steady future work. Much has come out of the research already. Our research patients are accepted only through their medical attendants, who have shown a willing and cordial faith in us, as I may venture, perhaps, to illustrate by the case of Dr. R. C. Brown, of Preston, who is providing us with one of our present research staff, and has equipped us with a costly $x$-ray instalment. It is in this spirit of hope and confidence that a large body of practitioners in this country are welcoming this (if I may quote your own words) "unique institution," begun by "a small band of enthusiasts, of whom the most prominent was a well-known pathologist." We in Cambridge must not allow Mr. Strangeways' invincible modesty and reticence to conceal his name and indefatigable labours on all occasions; nor pretend to assume honours which are his. On this one occasion, at any rate, I will dare his resentment by mentioning his name.

Yours truly,
Clifford Allbutt.

St. Radegvnd's, Cambridge, December 14, 1908.

